# Grey and White Matter Changes across the Amyotrophic Lateral Sclerosis-Frontotemporal Dementia Continuum

**DOI:** 10.1371/journal.pone.0043993

**Published:** 2012-08-29

**Authors:** Patricia Lillo, Eneida Mioshi, James R. Burrell, Matthew C. Kiernan, John R. Hodges, Michael Hornberger

**Affiliations:** 1 Neuroscience Research Australia, Sydney, Australia; 2 School of Medical Sciences, University of New South Wales, Sydney, Australia; 3 ARC Centre of Excellence in Cognition and its Disorders, Sydney, Australia; University Of Cambridge, United Kingdom

## Abstract

There is increasing evidence that amyotrophic lateral sclerosis (ALS) and frontotemporal dementia (FTD) lie on a clinical, pathological and genetic continuum with patients of one disease exhibiting features of the other. Nevertheless, to date, the underlying grey matter and white matter changes across the ALS-FTD disease continuum have not been explored. In this study fifty-three participants with ALS (n = 10), ALS-FTD (n = 10) and behavioural variant FTD (bvFTD; n = 15) as well as controls (n = 18), underwent detailed clinical assessment plus structural imaging using voxel-based morphometry (VBM) and diffusion tensor imaging (DTI) analysis of magnetic resonance brain imaging to examine grey and white matter differences and commonalities across the continuum. Importantly, patient groups were matched for age, education, gender and disease duration. VBM and DTI results showed that changes in the ALS group were confined mainly to the motor cortex and anterior cingulate as well as their underlying white matter tracts. ALS-FTD and bvFTD showed widespread grey matter and white matter changes involving frontal and temporal lobes. Extensive prefrontal cortex changes emerged as a marker for bvFTD compared to other subtypes, while ALS-FTD could be distinguished from ALS by additional temporal lobe grey and white matter changes. Finally, ALS could be mainly distinguished from the other two groups by corticospinal tract degeneration. The present study shows for the first time that FTD and ALS overlap in anterior cingulate, motor cortex and related white matter tract changes across the whole continuum. Nevertheless, frontal and temporal atrophy as well as corticospinal tract degeneration emerged as marker for subtype classification, which will inform future diagnosis and target disease management across the continuum.

## Introduction

Amyotrophic lateral sclerosis (ALS) and Frontotemporal dementia (FTD) are multisystem neurodegenerative disorders [1], [2], which overlap at a clinical and pathological level [3], [4], [5]. Whilst a proportion of patients presenting with ALS manifest cognitive and behavioral changes which may be severe enough in some instances to reach criteria for frank FTD (10–15%) [6], [7], a subgroup of patients with FTD develops features of ALS (15%) [8]. This continuum has been reinforced on two levels: i) pathological - TAR DNA binding protein 43 kD (TDP-43) is the principal protein inclusion in ALS and in a subgroup of FTD cases [9], [10]; and recently ii) genetic – by identifying a unique expansion in the intron of C9ORF72 on chromosome 9 (9p21) in families affected by ALS, FTD or ALS-FTD.

The C9ORF72 expansion was found in almost a quarter of cases with familial ALS and 12% of the cases with familial FTD [11], [12]. Patients with ALS and the C9ORF72 repeat expansion presented disease duration shorter than sporadic cases but with clinical features similar to the group without the mutation [13], [14], [15]. One study reported a higher frequency of bulbar cases associated with C9ORF72 repeat expansion [13]. Interestingly, ALS symptoms were most commonly found in the behavioural variant (bvFTD) subtype of FTD [16], [17], [18], [19], whereas ALS symptoms in the language variants (progressive non-fluent aphasia: PNFA; semantic dementia: SD) were infrequent [17], [18]. One of the studies found a high proportion of patients presenting with psychotic symptoms, characterised by florid delusions [19]. Similar to the sporadic FTD and ALS, the average age of presentation was in the fifties, but there was a wide spread of presentation age (32–76 years old) [16], [17], [18], [19], [20].

Despite the clinical, genetic and pathological overlap, atrophy correlates across the continuum have been rarely explored. ALS patients show atrophy of frontotemporal brain regions in addition to motor cortical changes [21], although motor cortex atrophy has been not as consistently observed as other cortical changes [22]. Similarly, FTD patients show a consistent pattern of grey matter atrophy in frontal lobes and temporal poles with subtypes varying in the degree of frontal, temporal and insula atrophy. Very few studies have investigated atrophy in patients with ALS-FTD overlap. As expected, patients with ALS-FTD have atrophy of frontotemporal lobes and hyperintensity of subcortical white matter in medial anterior temporal lobe, similar to that seen in patients with pure FTD [23]. A comparison of ALS and ALS-FTD patient groups using voxel-based morphometry (VBM) showed a similar pattern of grey matter atrophy involving bilateral motor and premotor cortices, superior, middle and inferior frontal gyri, superior temporal gyri, temporal poles and left posterior thalamus that was, as predicted, greater in the ALS-FTD group [22], [24]. Finally, a study of structural MRI from 5 family members affected with FTD-ALS linked to chromosome 9, showed loss of grey and white matter more prominent in frontal than temporal lobes [25].

To our knowledge, no neuroimaging study to date has investigated the grey and white matter changes in the whole ALS-FTD continuum (ALS, ALS-FTD, behavioural variant FTD). The current study set out to investigate atrophy and white matter changes between the syndromes. We hypothesised a gradation of prefrontal atrophy, with bvFTD being worst affected, followed by ALS-FTD and finally ALS. By contrast, an inverse grading pattern of atrophy was expected for the motor cortex, with more marked atrophy in ALS, followed by ALS-FTD and bvFTD. Similarly, white matter tract underlying the cortical atrophy should be affected, but we expected more corticospinal tract degeneration in ALS and ALS-FTD corroborating their ALS symptoms.

## Methods

### Case Selection

A total of 53 subjects participated. Ten patients with diagnosis of ALS (classified as definite or probable ALS, according with El Escorial Criteria Revised) [26] were recruited from a specialist ALS Multidisciplinary clinics in Sydney. The criteria required the demonstration of clinical or electromyographic evidence of lower motor neuron dysfunction in regions with concomitant upper motor neuron signs. ALS was diagnosed when combined lower and upper motor neuron dysfunction was identified in bulbar and spinal-innervated regions. Respiratory function measured by forced vital capacity (FVC) was above 70% and there was no evidence of nocturnal hypoventilation. Fifteen patients with a clinical diagnosis of bvFTD, who attended the FTD Research Clinic (FRONTIER), were also identified. All bvFTD patients met current consensus criteria for probable bvFTD with progressive deterioration of behaviour and/or cognition, including disinhibition, apathy, loss of empathy, perseveration, dietary changes, executive dysfunction and frontal and/or anterior temporal atrophy on MRI [27]. None of the bvFTD patients manifested symptoms of ALS. The ALS and bvFTD patients were contrasted against 10 patients with a diagnosis of ALS-FTD, who met criteria for both diseases (upper and lower motor neuron signs as well as behaviour and/or cognition deterioration). None of the patients had additional neurological disease or previous mental illness. In addition, 18 healthy control participants were included in the study. Age, gender, education, and disease duration were matched for the patient groups. All testing and scanning was performed at the first clinic visit for all patients.

### Ethics Statement

The research was conducted according to the principles expressed in the Declaration of Helsinki. Ethics approval was obtained from the Human Research Ethics Committee of South Eastern Sydney/Illawarra Area Health Service (HREC 10/126) and the University of New South Wales Human Research Ethics Advisory panel D (Biomedical, ref. #10035). Patient or family written consent was obtained from each participant.

### Cognitive and Behavioural Screening

The Addenbroke’s Cognitive Examination Revised (ACE-R) is a brief, sensitive and specific battery to detect early cognitive impairment and dementia. It contains 5 subscales: attention & orientation, memory, fluency, language, visuospatial, with higher scores denoting preserved cognitive abilities [28]. We employed the total score of the ACE-R in the analysis, which has a maximum score of 100. A score of 88 out of 100 or below suggests cognitive impairment, with 94% sensitivity and 89% specificity.

Changes in behaviour of patients were measured with the Cambridge Behavioural Inventory Revised (CBI-R) [29]. The CBI-R is a 45-item carer questionnaire which measures neuropsychiatric symptoms (challenging behavior, motivation, mood, abnormal beliefs, stereotypical behaviour and memory) and daily activities (self-care, eating and sleep) in dementia. The CBI-R rates the frequency of any particular behaviour on a scale of 0–4. A score of zero denotes no impairment, a score of 1 an occasional occurrence (a few times per month), 2 a repeated occurrence (a few times per week), 3 a daily occurrence, and 4 constant occurrence; the latter two scores signifying a severe behavioural deficit. Thus, a higher score is indicative of more behavioural dysfunction. The results of the CBI-R have been validated against the Neuropsychiatric Inventory (NPI). We report the total CBI-R score, which has a maximum score of 180.

### Behavioural Analyses

Data were analyzed using IBM SPSS 20.0. A priori, variables were plotted and checked for normality of distribution by Kolmogorov-Smirnov tests. Parametric demographic (age, education), neuropsychological (ACE-R) and behavioral (CBI-R) data were compared across the four groups (ALS, bvFTD, ALS-FTD and controls) via one-way ANOVAs followed by Tukey HSD post-hoc tests. Variables revealing non-normal distributions were log transformed and the appropriate log values were used in the analyses, but Table 1 reports their original values to facilitate clinical interpretation. Variables showing non-parametric distribution after log transformation were analysed via Chi-square (gender) and Kruskal-Wallis & Mann-Whitney U (disease duration) tests.

**Table 1:** Demographics, cognition and behaviour - Comparison on demographics and cognitive tests across ALS, bvFTD and ALS-FTD groups.

	bvFTD n = 15	ALS-FTD n = 10	ALS n = 10	Controls n = 18	*F* -values
Age (mean, SD) years	61.7 (7.1)	64.2 (7.0)	58 (10)	64.8 (5.3)	NS
Education (mean, SD) years	12 (3.2)	14 (4.2)	14.5 (3.2)	13.6 (2.5)	NS
Gender (M/F)	11/4 (2.8)	6/4 (1.5)	7/3 (2.3)	9/9 (1.0)	NS
Disease duration, years (median, interquartile range)	3.3 (1.8–5.6)	3.0 (1.8–4.4)	3.0 (1.1–5.0)	–	NS
ACE-R (total score; 0–100)	67.8 (17.6)	66.3 (14.2)	87.1 (10)	95.4 (2.9)	***
CBI-R (total score; 0–180)	72.7 (30.7)	46.6 (23.7)	34.7 (16.8)	7.2 (7.7)	***

F-values indicate significant differences across groups; Tukey post hoc tests compare differences between group pairs.

*** p<0.001; NS = non-significant.

Kruskal-Wallis Test and Mann-Whitney U test were applied for disease duration.

ALS = amyotrophic lateral sclerosis; ALS-FTD = ALS with frontotemporal dementia; bvFTD = behavioural variant frontotemporal dementia.

ACE-R = Addenbrooke’s Cognitive Examination revised; CBI-R = Cambridge Behavioural Inventory Revised.

### Imaging Acquisition

Patients and controls were scanned on a 3T Philips MRI scanner. T1-weighted acquisition: coronal orientation, matrix 256×256, 200, 1×1 mm^2^ in-plane resolution, slice thickness 1 mm, TE/TI = 2.6/5.8 ms.

DTI-weighted acquisition: 32 gradients, TR/TE/TI: 8400/68/90 ms, b-value = 1000 s/mm^2^, 55 2.5 mm horizontal slices, end resolution: 2.5×2.5×2.5 mm^3^, 96×96 matrix; repeated 2 times).

### Voxel-based Morphometry Analysis

Voxel based morphometry was conducted on the three dimensional T1-weighted scans using the FLS-VBM toolbox in the FMRIB software library package (http://www.fmrib.ox.ac.uk/fsl/).

In a first step, brain extraction was performed on all scans using the BET algorithm [30] in FSL using a fractional intensity threshold of 0.22. Brain extraction was checked visually for all scans so that no brain matter was excluded. Similarly, the scans were checked for whether any non-brain matter (e.g. skull, dura mater, optic nerve) were still present in the brain extracted images. If non-brain matter was visually detected or brain matter was falsely excluded, the BET algorithm for that scan was repeated by changing the fractional intensity threshold to give smaller or larger brain outline estimates.

A study-specific grey matter template was then created by including 10 scans of each group (total n = 40). The same number of scans across groups was used to avoid any bias during the registration step (i.e. favouring one group) while at the same time representing all included groups equally. The template scans were then registered to the Montreal Neurological Institute Standard space (MNI 152) using non-linear *b-spline* representation of the registration warp field resulting in study-specific gray matter template at 2×2×2 mm3 resolution in standard space.

At the same time, all brain extracted scans were also processed with the FMRIB’s Automatic Segmentation Tool (FAST v4.0) [31] to achieve tissue segmentation of i) grey matter, ii) white matter and iii) CSF via a hidden Markov random field model and an associated Expectation-Maximization algorithm. The FAST algorithm also corrected for spatial intensity variations such as bias field or radio-frequency inhomogeneities in the scans, resulting in partial volume maps of the scans.

In a next step, the gray matter partial volume maps were then non-linearly registered to the study-specific template via non-linear *b-spline* representation of the registration warp and were modulated by dividing them by the Jacobian of the warp field to correct for the contraction/enlargement due to the non-linear component of the transformation [32].

Finally, the normalised and modulated grey matter maps were smoothed with an isotropic Gaussian kernel (standard deviation = 3 mm; full width half maximum = 8 mm).

The statistical analysis was performed by employing a voxel-wise general linear model. Significant clusters were formed by employing the threshold-free cluster enhancement (TFCE) method [33]. The TFCE method is a cluster-based thresholding method which does not require the setting of an arbitrary cluster forming threshold (e.g. t,z<4), instead it takes a raw statistics image and produces an output image in which the voxel-wise values represent the amount of cluster-like local spatial support. The TFCE image is then turned into voxel-wise p-values via permutation testing. We employed a permutation-based non-parametric testing with 5000 permutations [34].

All group comparisons were tested for significance at *p*<0.05, corrected for multiple comparisons via Family-wise Error correction across space, except for the ALS vs. Controls contrast, which did not survive FWE correction and was tested at a significance level of p<0.001, uncorrected and a cluster threshold of 20 contiguous voxels.

### Diffusion Tensor Imaging (DTI) Analysis

In a first step, the two DTI sequences were averaged for each participant, visually checked for field inhomogeneity distortions. They were further corrected for eddy current distortions (i.e. stretches and shears induced by the gradient coils during image acquisition) by using affine registration to the b0 reference volume.

The diffusion tensor models were then fitted at each voxel via the FDT toolbox in FSL (http://www.fmrib.ox.ac.uk/fsl/fdt/index.html), resulting in maps of three eigenvalues (L1, L2, L3) which allowed calculation of fractional anisotropy (FA) maps for each subject. FA maps for all subjects were visually checked to identify errors in the fitting of the eigenvalues. In particular, corpus callosum, corticospinal tract and inter-hemispheric commissure were visually checked for fibre orientation information as they have some of the most consistent white matter orientations in the brain.

Tract-Based Spatial Statistics (TBSS) [35] from FSL were used to perform a skeleton-based analysis of white matter FA. FA maps of each individual subject were nonlinear coregistered using FNIRT [32], [36] to the MNI standard space of the FMRIB58_FA template and were visually checked for any registration errors. The template was subsampled at 1×1×1 mm^3^ due to the coarse resolution of native DTI data (i.e. 2.5×2.5×2.5 mm^3^). After image registration, FA maps were averaged to produce a group mean FA image.

A skeletonization algorithm [35] was applied to the group mean FA image to define a group template of the lines of maximum FA, assumed to correspond to centers of white matter tracts. The resulting binary skeleton mask defines the set of voxels used in all subsequent processing. Next a distance map is created from the skeleton mask. FA values for each individual subject were then projected onto this group template skeleton.

Similar to the VBM analysis, the skeletonized FA data was then statistically tested via a voxel-wise general model. Significant clusters were formed by employing the threshold-free cluster enhancement (TFCE) method as described in the VBM analysis. Clusters were tested using permutation-based non-parametric testing with 5000 permutations.

**Figure 1:**
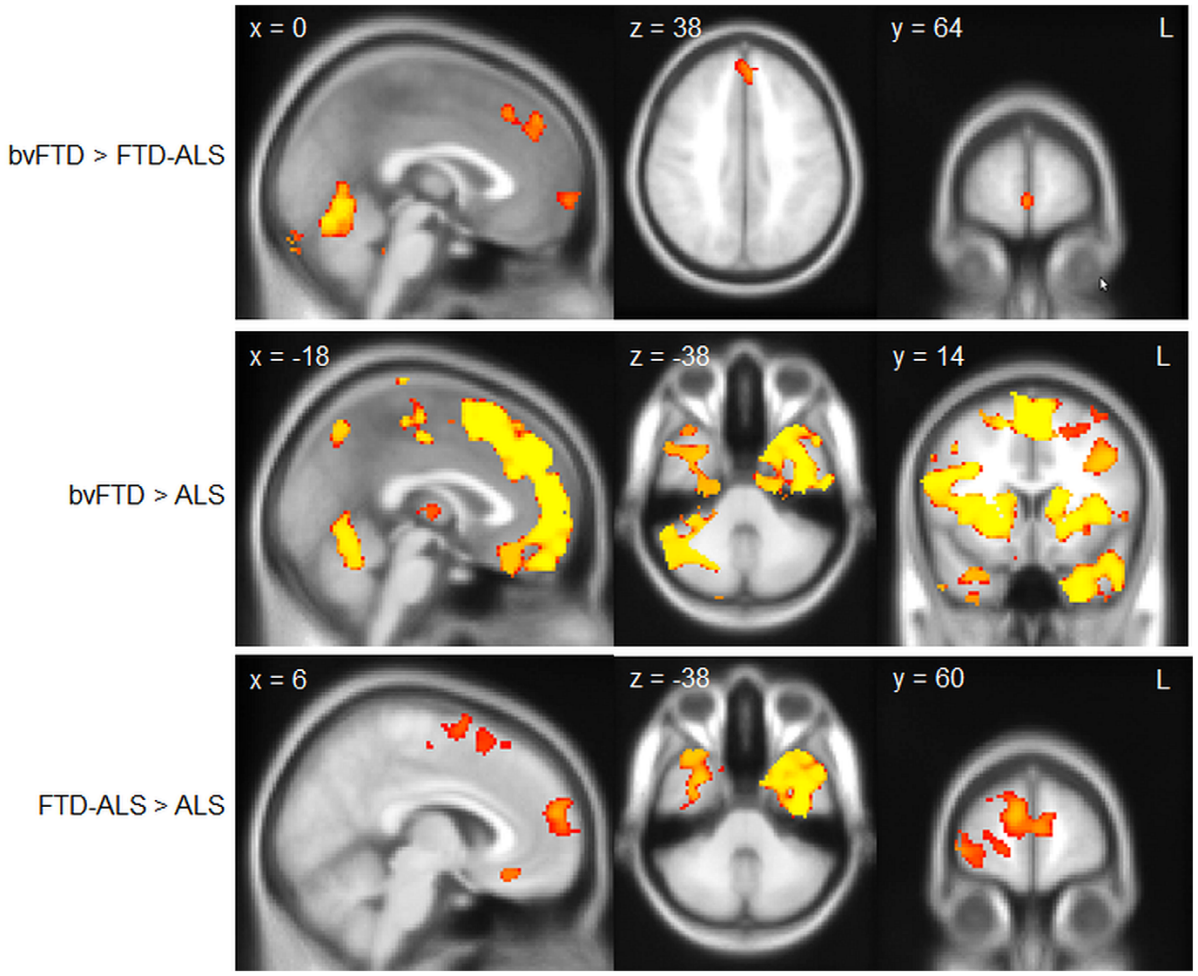
Unique grey matter atrophy across patient groups. Voxel-based morphometry analysis showing regions of unique brain atrophy across groups. Clusters are overlaid on the MNI standard brain (t = 2.41). Coloured voxels show regions that were significant in the analyses for p<0.05 FWE corrected.

Clusters reported have significance at p<0.05, corrected for multiple comparisons across via family-wise error correction space for patient vs. control comparisons. The inter-patient comparisons were thresholded at p<.001, uncorrected.

The overlap analyses for the VBM and DTI data were by created by thresholding the individual patient vs. control contrasts at p<.001, uncorrected in a first step. This was followed by a multiplication of the contrasts which resulted in inclusive or overlap masks across groups.

**Figure 2:**
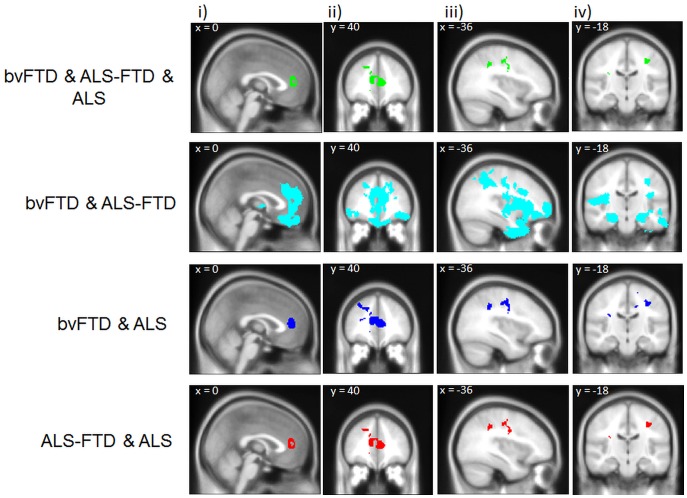
Overlap grey matter atrophy across patient groups. Voxel-based morphometry analysis showing region of brain atrophy overlap; i) and ii) show sagittal and coronal views of the anterior cingulate atrophy cluster; iii) and iv) show sagittal and coronal views of the motor cortex atrophy cluster. Clusters are overlaid on the MNI standard brain (t = 2.41).

## Results

### Demographics

No significant differences were identified for any of the demographic variables (age, gender and education) across patient groups and the control cohort (p>.1, Table 1). Importantly, disease duration did not differ between the ALS, ALS-FTD and bvFTD groups (p>.1).

### Cognitive and Behavioral Profile

Comparison of the ACE-R score showed a significant difference between groups [F (3,48) = 19.9, p<.001]. Post hoc comparisons using Tukey HSD tests indicated that both the ALS-FTD and bvFTD groups performed significantly worse than controls (p<.001) but there was no significant difference between ALS and controls (p>1), and ALS patients performed better than ALS-FTD and bvFTD groups (p’s<.01) (see Table 1). Finally, bvFTD and ALS-FTD patients were not different from each other (p>1).

**Figure 3:**
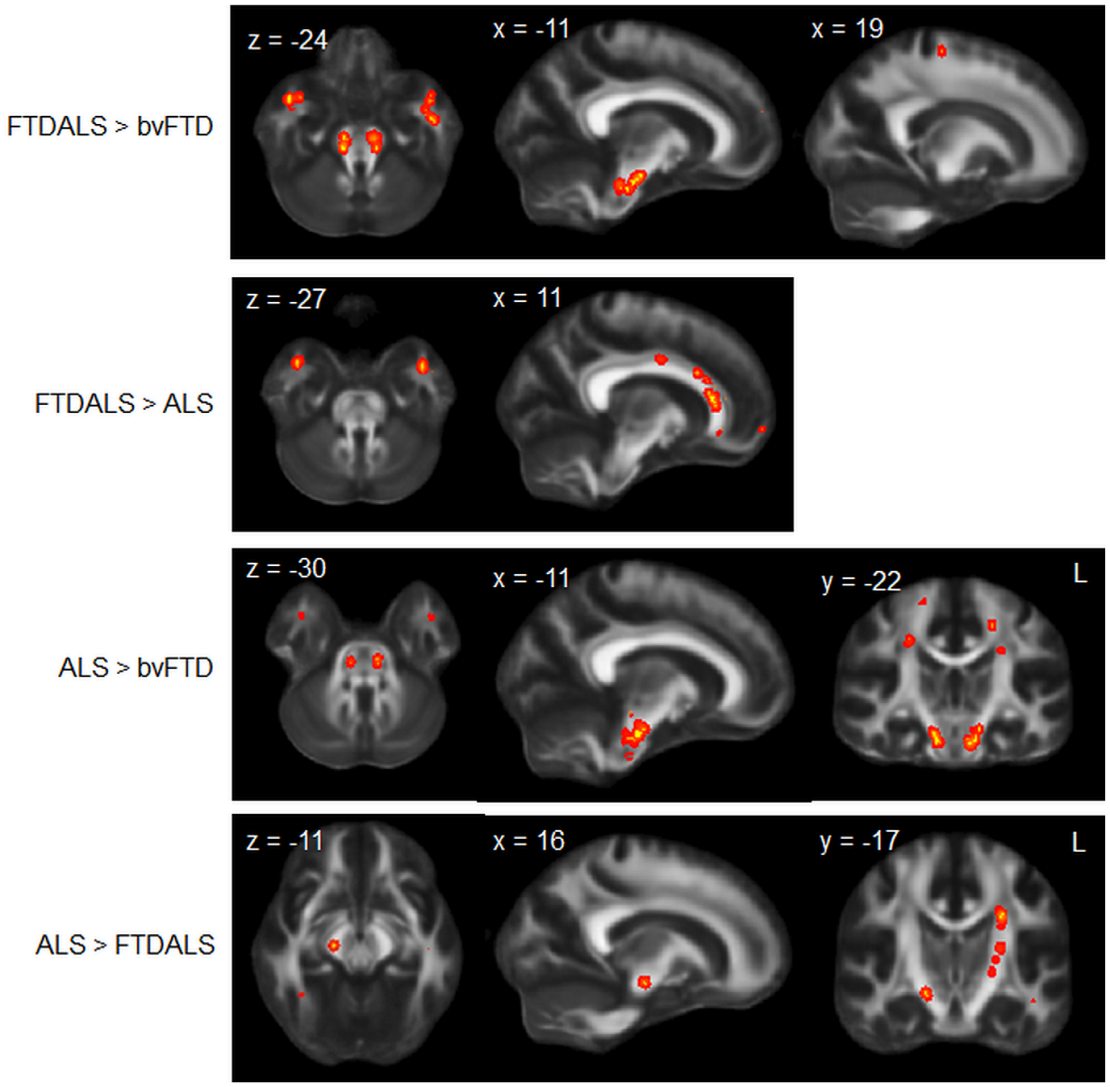
Unique white matter changes across patient groups. Diffusion tensor imaging analysis showing regions of unique white matter changes across groups. Clusters are overlaid on the MNI standard brain (t = 2.41). Coloured voxels show regions that were significant in the analyses for p<0.05 FWE corrected.

There was also a significant difference across group on the CBI-R total score [F (3,46) = 29.01, p<.001). Controls had lower score than bvFTD (p<.001), ALS-FTD (p<.001) and ALS (p<.01); whereas patients with bvFTD had higher scores than ALS-FTD (p<.05) and ALS (p<.001) groups. No significant difference was found between ALS-FTD and ALS (p>.1).

### Voxel-based Morphometry

#### Differences with controls

In comparison to controls, bvFTD patients showed marked gray matter atrophy affecting the frontal pole, orbitofrontal area, anterior cingulate, superior frontal gyrus, premotor and motor cortices, anterior insula, temporal poles, thalamus and striatum bilaterally (Figure S1A). The pattern of atrophy in ALS-FTD was very similar to that found in bvFTD, involving frontotemporal regions but with less involvement of frontal pole and superior frontal gyrus (Figure S1B). In contrast, ALS patients showed significant gray matter atrophy in anterior cingulate, paracingulate and motor cortices (precentral gyrus) only (Figure S1C).

**Figure 4:**
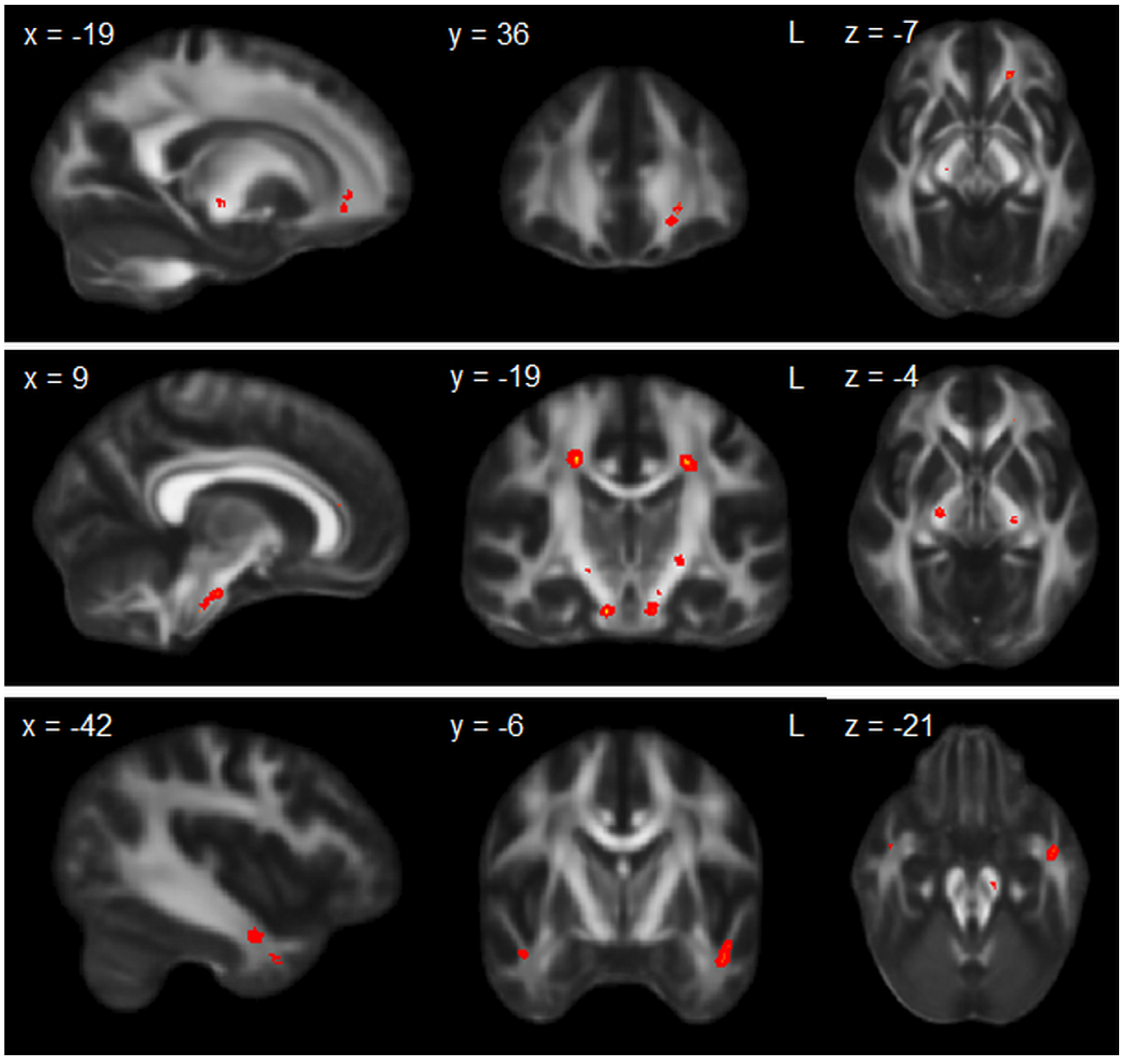
Overlap white matter changes across patient groups. Diffusion tensor imaging analysis showing regions of unique white matter changes across groups. Clusters are overlaid on the MNI standard brain (t = 2.41). Coloured voxels show regions that were significant in the analyses for p<0.05 FWE corrected.

#### Differences between patient groups

To investigate patterns of atrophy unique to each group we compared all the patient groups with each other. A contrast between bvFTD and ALS-FTD (Figure 1) showed more atrophy in superior frontal and paracingulate gyrus for bvFTD and no significant differences for the temporal lobes. The reverse contrast did not reveal any significant differences, i.e. no additional atrophy in ALS-FTD compared to bvFTD.

A comparison of ALS and bvFTD showed greater atrophy including frontal pole, superior frontal gyrus, anterior cingulate and paracingulate gyrus, insular cortex and temporal lobes in bvFTD (Figure 1). No areas showed greater atrophy in ALS than bvFTD group.

Finally, in comparison to ALS cases, ALS-FTD cases had greater atrophy in frontal and temporal lobes (Figure 1). The reverse contrast revealed no significant differences between ALS and ALS-FTD patients.

**Figure 5:**
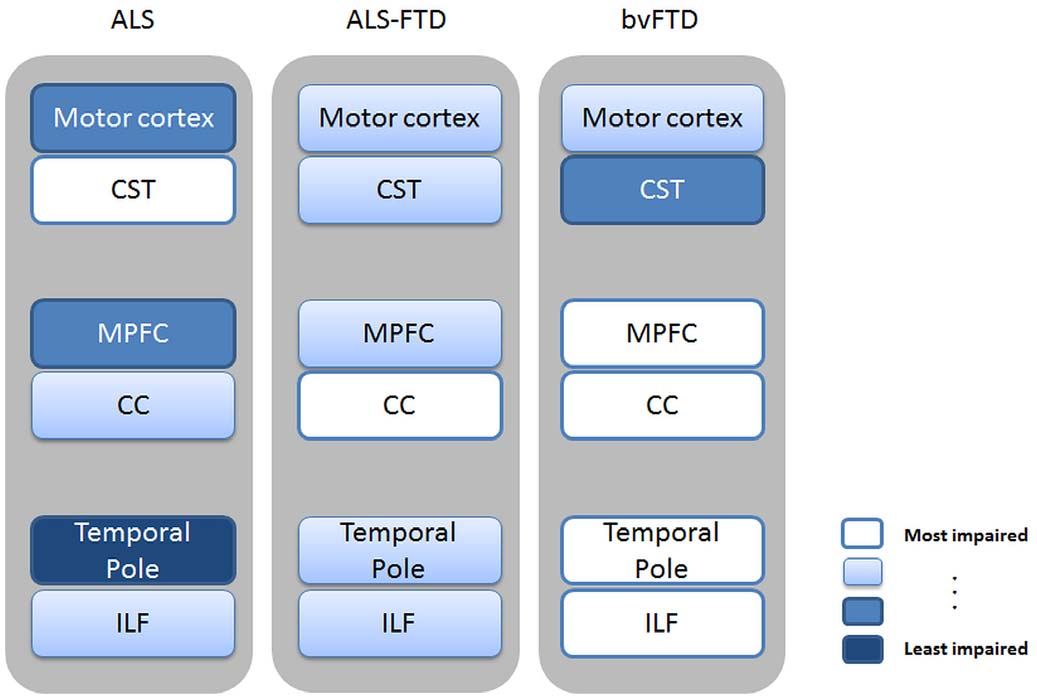
Schematic summary of main findings. Summary of main grey matter regions and their underlying white matter tracts across patient groups. Shading of text boxes indicates severity of impairment based on the imaging findings with lighter boxes indicating higher impairment and darker boxes indicated lesser impairment. CST = corticospinal tract; MPFC = medial prefrontal cortex; CC = corpus callosum; ILF = inferior longitudinal fasciculus.

#### Overlap between patient groups

In a set of final VBM analyses, we inclusively masked the atrophy across patients to reveal atrophy overlap between ALS, ALS-FTD and bvFTD. In a first step, we conducted an analysis across the whole continuum (ALS, ALS-FTD and bvFTD) to reveal common areas of gray matter atrophy. Atrophy in anterior cingulate and paracingulate gyrus, frontal and middle frontal gyri (Figure 2 i & ii), motor cortex (precentral gyrus) and supramarginal gyrus (Figure 2 iii & iv) was common to all patient groups.

Pair-wise atrophy overlap between bvFTD and ALS-FTD showed large areas of common prefrontal cortex, motor cortex and temporal lobe atrophy, as shown in Figures 2 i-iv.

Finally, a very similar pattern of common gray matter atrophy was found between bvFTD and ALS (Figures 2 i-iv) and between ALS-FTD and ALS (Figures 2 i-iv), including anterior cingulate, motor cortex and supramarginal gyrus, which resembles the overlap found between the three diseases.

### Diffusion Tensor Imaging (DTI) Analysis

#### Differences with controls

In comparison to controls, bvFTD showed substantial forceps minor, anterior corpus callosum, anterior inferior longitudinal fasciculus and corticospinal tract degeneration (Figure S2).

Similarly, ALS-FTD patients showed white matter degeneration in the same tracts as bvFTD but to a lesser degree in the forceps minor and anterior corpus callosum. They further showed a more anterior part of the inferior longitudinal fasciculus being affected and more substantial corticospinal tract degeneration (Figure S2).

As expected, ALS patients showed the most substantial changes in the corticospinal tract compared to controls, while only small changes were observed in the forceps minor, anterior corpus callosum as well as the inferior longitudinal fasciculus (Figure S2).

#### Differences between patient groups

Comparison between patient groups showed more substantial white matter degeneration for bvFTD in forceps minor, anterior corpus callosum as well as the inferior longitudinal fasciculus compared to ALS-FTD and ALS. More interestingly, however was that ALS-FTD and ALS patients showed also more white matter degeneration than bvFTD. In particular, corticospinal tracts in the brainstem and the motor cortex were more affected in ALS and ALS-FTD than in bvFTD (Figure 3). In addition, both patient groups showed more temporal pole white matter degeneration than bvFTD. Contrasting the two ALS subtypes with each other revealed that ALS-FTD had more forceps minor and anterior corpus callosum, as well as inferior longitudinal fasciculus degeneration than ALS, while ALS patients only showed more corticospinal tract degeneration in comparison to ALS-FTD (Figure 3).

#### Overlap between patient groups

Finally, overlap DTI analyses showed that the whole continuum (ALS, ALS-FTD and bvFTD) showed degeneration in parts of the corticospinal tract, inferior longitudinal fasciculus and anterior corpus callosum, reflecting the overlap grey matter changes (Figure 4).

## Discussion

The grey and white matter findings in bvFTD replicate previous studies, showing substantial involvement of ventromedial prefrontal cortex, insula, temporal and striatal regions and their underlying white matter tracts [37], [38]. Of particular interest was the finding of motor cortex atrophy and corticospinal degeneration in bvFTD, in keeping with a recent report of TMS motor hyperexcitability in a high proportion of FTD patients [39].

Motor cortex atrophy was also observed in the ALS patients, though, contrary to our predictions, this was not significantly greater in ALS compared to the other patient groups. Surprisingly, motor cortex atrophy has not been consistently observed in ALS neuroimaging studies, with earlier findings showing grey matter atrophy in frontal and temporal areas but no volume reduction in primary motor cortex [22]. A recent meta-analysis, including 5 studies and comprising data of 84 ALS patients, showed that around 25% of ALS patients had motor cortex atrophy [40]. By contrast, corticospinal degeneration was significantly more affected in ALS than the other disease groups. The other region consistently atrophied in ALS compared to controls in our study was the anterior cingulate and the underlying anterior corpus callosum tract, which replicates a previous study [41]. Recent imaging studies in ALS using voxel-based morphometry (VBM) and diffusion tensor imaging (DTI), have also shown that the severity of apathy correlated with atrophy in the orbitofrontal, mesial and dorsolateral prefrontal cortices, particularly on the right side [42], [43].

Finally, our findings of widespread frontal, temporal, motor and subcortical changes in ALS-FTD corroborate pathological [44] and grey matter neuroimaging [45] [24] findings.

To our knowledge, this is the first study investigating white matter changes in FTD-ALS, showing complementary white matter degeneration to the cortical regions affected, but also substantial corticospinal tract degeneration (Figure 5).

Unique grey and white matter changes were also observed for all patient groups. Patients with bvFTD had substantially more prefrontal, temporal and striatal atrophy than ALS and greater prefrontal cortex atrophy in comparison to ALS-FTD. Similarly, prefrontal and temporal white matter tracts were more impaired in bvFTD compared to the other two subtypes. In comparison to those with pure ALS, patients with ALS-FTD showed more grey atrophy in the temporal and insula regions, while the degree of prefrontal cortex atrophy was similar. Interestingly, however, was that white matter changes in ALS-FTD were more substantial in temporal regions compared to both bvFTD and ALS. At the same time, ALS-FTD patients showed more white matter degeneration in corticospinal and prefrontal tracts compared to bvFTD and ALS, respectively. Finally, no grey matter atrophy was found in ALS compared to bvFTD and ALS-FTD. Strikingly, however, was that ALS patients showed more white matter changes compared to bvFTD and ALS-FTD, with corticospinal tracts being particularly more affected in this condition. The grey matter findings have been only previously shown on a pathological level, where ALS vs. ALS-FTD and FTD (FTLD-U) showed motor, midfrontal, temporal and parietal atrophy which varied in extent across the 3 groups, with the ALS group having a milder degree of pathology in comparison to ALS-FTD and FTLD-U [44]. This also is supported by the clinical continuum in which the cognitive and behavioural profiles of ALS mirror those seen in bvFTD, but with more marked deficits in the latter group. [5] [46].

Similarly, pair-wise neuroimaging comparisons between FTD and ALS-FTD have shown a graded pattern of atrophy with FTD showing a more widespread pattern of atrophy than ALS-FTD [24], [45], further corroborating our findings. To our knowledge, the white matter findings have not been shown across the continuum so far.

More importantly, overlap analyses between the three subtypes revealed that anterior cingulate, premotor and motor areas as well as their underlying white matter tracts were equally affected across all groups. One speculative suggestion relates to the speed of evolution of the common cortical TDP-43 pathology leading to rapid dissolution of cortico-cortico connections that might set the stage the clinical presentation with motor symptoms and behavioural manifestations.

While anterior cingulate and premotor atrophy are not surprising findings in bvFTD, motor cortex atrophy and corticospinal tract degeneration has only recently attracted attention with pathological studies indicating involvement of motor cortex in FTD [47]. Similarly, a recent study employing clinical and neurophysiological (i.e. threshold tracking transcranial magnetic stimulation) methods showed that FTD and ALS patients had abnormal cortical excitability, confirming dysfunction of the motor system in FTD [39].

Anterior cingulate and associate white matter tract degeneration is a hallmark of bvFTD [37], [38] and has been reported to be affected in ALS-FTD as well [24]. Neuroimaging studies have highlighted abnormalities in this region and adjacent white matter tracts in the corpus callosum in ALS [48]–[49]. Interestingly, anterior cingulate pathology has been associated with deficits in motivation (i.e. apathy) and stereotypical movements in bvFTD [50] and a recent study showed that anterior cingulate atrophy was closely related to the degree of apathy in ALS patients [43]. Importantly, apathy is the most prominent features in patients with ALS [51]. Clearly, the neural correlates of the behavioural dysfunction across the continuum need to be further investigated in the future.

Clinically, our findings reinforce the concept of a continuum between the three conditions not only on a pathological and genetic, but also on grey and white matter degeneration level, and explain the overlap in both behaviour and motor functions. In particular similar neural networks comprising motor cortical, medial prefrontal cortex and temporal pole regions and their afferent & efferents seem to be affected across the diseases. To date, however, very little is know how these neural networks interact functionally and whether damage to one network can affect other regions or networks involved. Neuroanatomically it has been known for a long time that all three regions show some connectivity and thus the overlap in symptoms and neuronal damage across the ALS-FTD continuum should be not surprising.

The shared changes of motor cortex and corticospinal tract explain why bvFTD, ALS-FTD and ALS patients can present with pyramidal motor symptoms, though they vary considerably in severity. Similarly, the changes in anterior cingulate and related white matter tracts may explain the presence of apathy, a pervasive symptom present across the spectrum [51]. The fact that anterior temporal lobe atrophy distinguished ALS-FTD from ALS is of particular interest and suggests that spread of pathology beyond the temporal lobe may predict the full-blown clinical picture of FTD in ALS patients. In addition, for ALS patients the discrepancy between variable grey matter but substantial white matter changes is of importance and highlights the fact that despite the same underlying pathology ALS and FTD differ considerably in which brain matter changes are observed (see also Figure 5).

Future studies, replicating our findings in larger samples as well as tracking atrophy and white matter changes longitudinally may further clarify subtype specific deficits across the ALS-FTD continuum. A critical issue in ALS is whether a subgroup of ALS patients have associated FTD from an early stage or alternatively whether atrophy spreads from the motor cortex to adjacent dorsolateral prefrontal cortex and temporal lobe structures at a later stage in the majority of ALS patients.

In conclusion, our study showed grey and white matter changes across ALS, ALS-FTD and bvFTD, identifying areas of difference and overlap between the three conditions, further corroborating the recent genetic overlap findings between the syndromes [11]–[12] [52]. This will inform future studies investigating the underlying symptoms across the ALS-FTD continuum, which in turn will provide better symptom characterisation and disease modifying therapies.

## Supporting Information

Figure S1 **Grey matter atrophy of patients compared to controls.** Voxel-based morphometry analysis showing brain area atrophy for **A**) bvFTD vs. controls, **B**) ALS-FTD vs. controls, and **C**) ALS vs. controls. Clusters are overlaid on the MNI standard brain (t = 2.41). Coloured voxels show regions that were significant in the analyses for p<0.05 FWE corrected, except for ALS vs. controls which was thresholded at p<.001, uncorrected, and a cluster threshold of 20 contiguous voxels.
(TIF)

Figure S2 **White matter changes of patients compared to controls.** Diffusion tensor imaging analysis showing white matter changes for **A)** bvFTD vs. controls, **B**) ALS-FTD vs. controls, and **C**) ALS vs. controls. Clusters are overlaid on the MNI standard brain (t = 2.41). Coloured voxels show regions that were significant in the analyses for p<0.05 FWE corrected, except for ALS vs. controls which was thresholded at p<.001, uncorrected, and a cluster threshold of 20 contiguous voxels.
(TIF)
