# Supplementary figures and images for: Grey and White Matter Changes across the Amyotrophic Lateral Sclerosis-Frontotemporal Dementia Continuum
Source: PLoS One. 2012 Aug 29;7(8):e43993. doi: 10.1371/journal.pone.0043993 (PMC3430626; doi:10.1371/journal.pone.0043993)

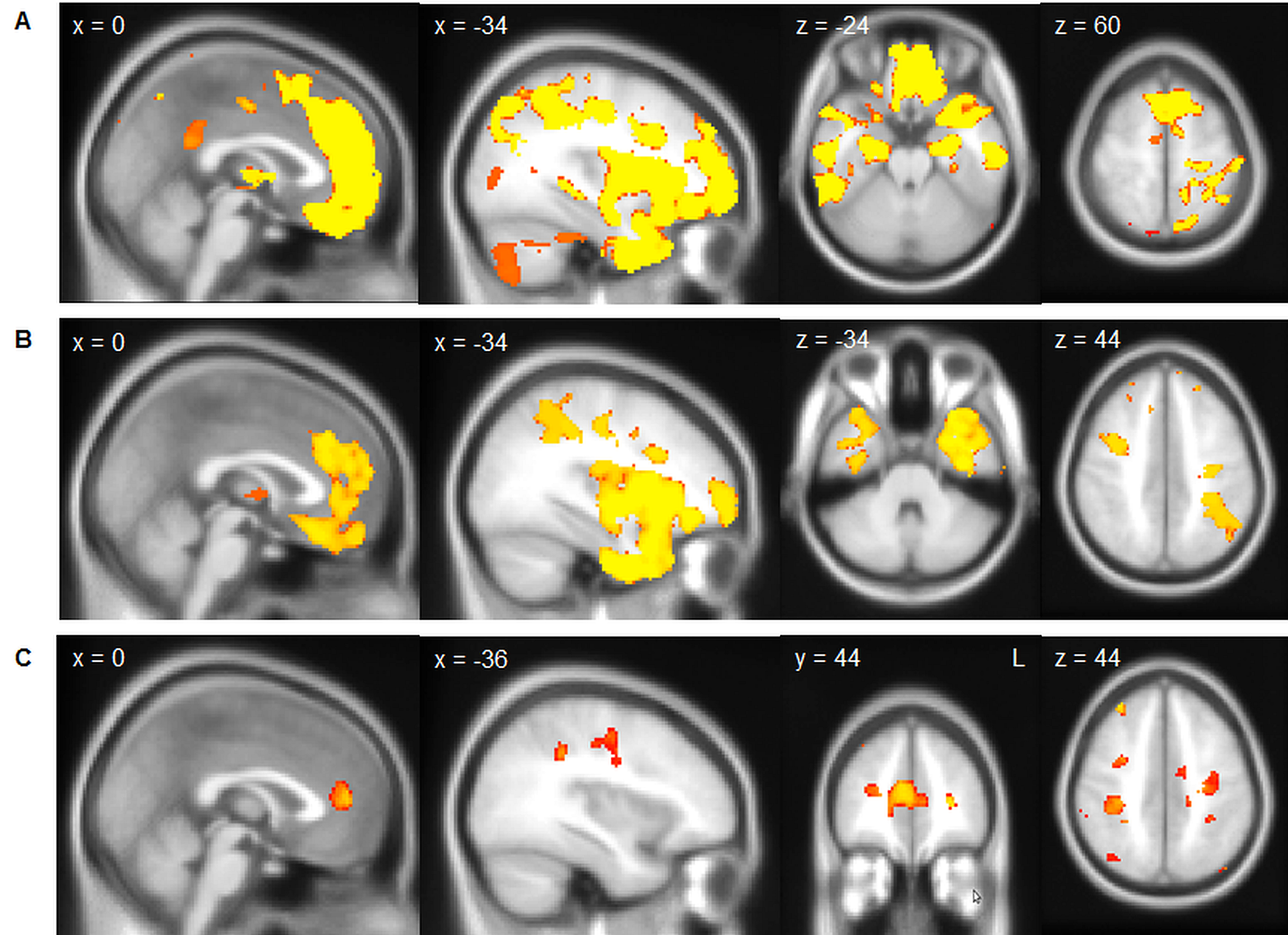

Supplement: Figure S1 — Grey matter atrophy of patients compared to controls. Voxel-based morphometry analysis showing brain area atrophy for A) bvFTD vs. controls, B) ALS-FTD vs. controls, and C) ALS vs. controls. Clusters are overlaid on the MNI standard brain (t = 2.41). Coloured voxels show regions that were significant in the analyses for p<0.05 FWE corrected, except for ALS vs. controls which was thresholded at p<.001, uncorrected, and a cluster threshold of 20 contiguous voxels. (TIF) [file pone.0043993.s001.tif]

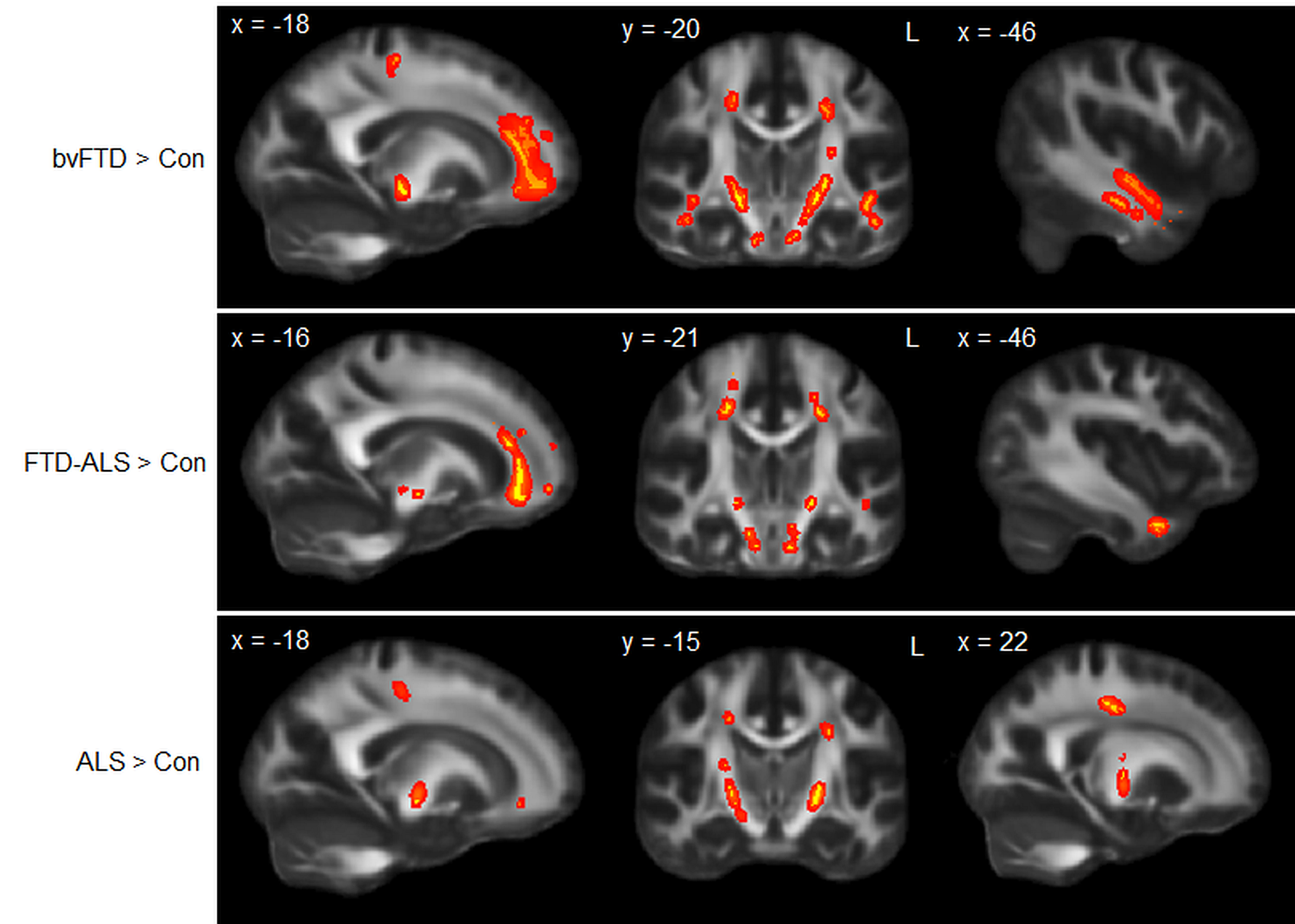

Supplement: Figure S2 — White matter changes of patients compared to controls. Diffusion tensor imaging analysis showing white matter changes for A) bvFTD vs. controls, B) ALS-FTD vs. controls, and C) ALS vs. controls. Clusters are overlaid on the MNI standard brain (t = 2.41). Coloured voxels show regions that were significant in the analyses for p<0.05 FWE corrected, except for ALS vs. controls which was thresholded at p<.001, uncorrected, and a cluster threshold of 20 contiguous voxels. (TIF) [file pone.0043993.s002.tif]
